# Lipschitz-Nonlinear Heterogeneous Multi-Agent Adaptive Distributed Time-Varying Formation-Tracking Control with Jointly Connected Topology

**DOI:** 10.3390/e27060648

**Published:** 2025-06-17

**Authors:** Ling Zhu, Yuyi Huang, Yandong Li, Hui Cai, Wei Zhao, Xu Liu, Yuan Guo

**Affiliations:** 1 School of Mechanical and Electrical Engineering, Qiqihar University, Qiqihar 161000, China; 02652@qqhru.edu.cn; 2 School of Computer and Control Engineering, Qiqihar University, Qiqihar 161006, Chinacaihui2000ch@icloud.com (H.C.);; 3 Heilongjiang Key Laboratory of Big Data Network Security Detection and Analysis, Qiqihar University, Qiqihar 161000, China; 4 School of Computer and Big Data, Heilongjiang University, Harbin 150080, China

**Keywords:** multi-agent system, switching communication topologies, adaptive control, time-varying formation tracking

## Abstract

This paper studies the problem of time-varying formation-tracking control for a class of nonlinear multi-agent systems. A distributed adaptive controller that avoids the global non-zero minimum eigenvalue is designed for heterogeneous systems in which leaders and followers contain different nonlinear terms, and which relies only on the relative errors between adjacent agents. By adopting the Riccati inequality method, the adaptive adjustment factor in the controller is designed to solve the problem of automatically adjusting relative errors based solely on local information. Unlike existing research on time-varying formations with fixed and switching topologies, the method of jointly connected topological graphs is adopted to enable nonlinear followers to track the trajectories of leaders with different nonlinear terms and simultaneously achieve the control objective of the desired time-varying formation. The stability of the system under the jointly connected graph is proved by the Lyapunov stability proof method. Finally, numerical simulation experiments confirm the effectiveness of the proposed control method.

## 1. Introduction

With the development of the times, research on multi-agent systems (MASs) has increased significantly, and they have been widely studied in fields such as unmanned aerial vehicle (UAV) collaboration, robot swarms, smart grids, and sensor networks. The research domain of MASs has become increasingly broad. For example, studies in multiple aspects, such as consensus control of MASs [1,2,3,4], cooperative control of heterogeneous MASs [5,6], and formation-tracking control of MASs [7,8], provide theoretical support for practical applications. Recent studies on consensus control have been carried out. For example, Reference [9] examines the consensus control of fractional-order MASs with reaction-diffusion terms, and References [10,11] investigates the consensus problem of estimating state-boundary control for fractional-order MASs and the consensus problem of modeling partial differential equation–ordinary differential equation systems through boundary control with directed topologies.

With the continuous deepening of research on consensus control problems for MASs, many scholars have found that formation control problems can be utilized to address consensus control issues. Reference [12] proposes an operator-based Newtonian trajectory optimization method to solve the problem of second-order, time-invariant optimal distributed formation tracking. Reference [13] employs adaptive optimal control parameters and neural networks to address the tracking control of optimal time-varying formation (TVF) under disturbances. Reference [14] presents a TVF control method that achieves specific formation shapes based on relative errors between agents, and has, consequently, attracted extensive attention from scholars in the field of TVF tracking control. Reference [15] investigates TVF tracking control for MASs with second-order integrator models under switching topologies and applies it to the practical application of quadrotor UAV flight. Reference [16] solves the formation control problem for a class of second-order MASs with communication delays based on a leader–follower approach. Reference [17] uses backstepping and simplified reinforcement learning methods to address a class of second-order uncertain MAS problems and employs an adaptive neural network state observer to handle unmeasurable state issues.

However, both linear and nonlinear integrators have limited generality; in other words, they are applied in specific scenarios. By contrast, nonlinear systems are more common in practice, making it more valuable to design MASs that can be widely applied to nonlinear dynamic models. References [18,19,20,21] all adopt leader–follower methods to study the consensus tracking control of MASs. Reference [22] proposes a class of MASs with multiple leaders to achieve TVF control. Reference [23] solves the problem of time-varying output formation control for heterogeneous MASs with multiple leaders under changing dynamic topologies by using distributed observers. Reference [24] addresses a sampled-data controlled MASs, completing TVF tracking control by designing a sampled-data controller with a larger sampling interval. However, it is evident that consensus control problems studied based on relative errors between agents and leader–follower methods are only special cases of formation control problems. In other words, most consensus control problems can be solved by formation control approaches. Additionally, References [22,24] all consider linear system models to achieve TVF, while References [23,25] design control laws for heterogeneous linear system models. Reference [26] proposes a consensus control method for heterogeneous nonlinear systems with arbitrary time-varying dynamics, where different agents contain distinct nonlinear terms. However, existing research on heterogeneous nonlinear systems remains limited. Furthermore, the control algorithms in References [22,23,24] do not achieve true distributed control, as they cannot complete formation control solely based on relative errors between adjacent agents. When facing large-scale MASs, these methods still require global topological information for processing, which is computationally challenging in practical scenarios. How to avoid global information and solve system problems using only local information remains a severe challenge. Reference [27] employs a low-gain feedback method to address adaptive TVF with input saturation, while Reference [28] uses observers to handle adaptive TVF with unilateral nonlinearities. Reference [29] considers adaptive TVF control under communication constraints. Reference [30] utilizes an adaptive feedback control method based on neural networks to solve formation control problems for a class of nonlinear MASs under directed graphs. Reference [31] solves a distributed optimal formation-tracking control problem using only relative information between agents and the cost functions of neighboring agents.

Nevertheless, in practical applications, the communication links of MASs are often affected by external environments and system failures, leading to communication interruptions in harsh environments or when agents fail. Additionally, the introduction of new agents may alter the existing connection structure, further increasing the time-varying nature of the communication network. These factors significantly complicate the control of MASs. Therefore, when designing collaborative control strategies for MASs, the key challenge is to rely on local information to design effective control mechanisms that maintain system synchronization despite issues such as communication interruptions, reconnections, and delays. Reference [32] uses a dynamic event-triggered mechanism to address communication resource overload and solves the formation control problem with communication delays caused by factors such as signals and bandwidth. Reference [33] proposes a finite-time output control algorithm combined with heterogeneous nonlinear systems to achieve formation control strategies. Reference [34] uses fuzzy control methods to address the impact of uncertain functions in uncertain systems and employs distributed optimization algorithms to achieve formation control objectives with time-varying delays. Inspired by the above literature, the innovations of this paper are as follows:

First, a heterogeneous MAS is constructed by combining leaders and followers with different nonlinear term structures. Unlike time-invariant formations, a desired TVF vector is designed to satisfy TVF conditions, ensuring that followers track the state trajectories of leaders while achieving the desired TVF.

Second, for the communication topology of heterogeneous nonlinear agents, unlike fixed topologies, the communication topology switches periodically. This ensures that the designed controller achieves the expected TVF and leader trajectory tracking under a jointly connected topological graph.

Third, the controller in this paper is fully distributed. By designing adaptive weight adjustment using only the relative errors between adjacent agents, it avoids the non-zero minimum eigenvalue constraint problem in global communication topologies to achieve TVF control.

## 2. Graph Theory and Problem Description

### 2.1. Graph Theory

Define G={V,E}, which describes an undirected graph, where $V=\{v_{1},v_{2},\ldots ,v_{n}\}$ represents the set of points of the agent in the graph G. Moreover, $E\subseteq \{\left(v_{i},v_{j}\right):v_{i},v_{j}\in V;i\neq j\}$ represents the set of edges in the undirected graph G, and $E_{ij}=\left(v_{i},v_{j}\right)$ represents the information flow between nodes $v_{i}$ and $v_{j}$ in the graph, which can interact with each other, and form an edge of G. A non-negative matrix $W=\left[w_{ij}\right]\in R^{N\times N}$ is defined as the adjacency matrix, where the elements $W=\{w_{ij}\}$ satisfy $E_{ij}\in E$; that is, $w_{ij}=1$ and vice versa $w_{ij}=0$. A diagonal matrix $F\in R^{N\times N}$ is represented as $F=\mathrm{diag}\left(b_{1},b_{2},b_{3},\ldots ,b_{n}\right)\in R^{N\times N}$, and represents the degree matrix. The *i*-th diagonal element is defined as $b_{i}=\sum_{j=1}^{N}w_{ij}$, so the Laplacian matrix of the graph G is defined as follows: L=F−W. When MASs consist of N agents and a leader, the leader can be described by vertex 0. Define a diagonal matrix $D=\mathrm{diag}\left\{d_{1},d_{2},\ldots ,d_{N}\right\}\in R^{N\times N}$, representing the adjacency matrix of the leader for information interaction between the leader and neighboring follower agents. If there is information flow, then $d_{i}=1$; otherwise $d_{i}=0$. Define the graph $\bar{G}$ on the vertex {0,1,2,…,N} on the graph, which is composed of the leader, the edges between the leader and the followers, the follower agents, and the graph G. Under the jointly connected topology, $H_{\sigma (t)}=L_{\sigma (t)}+D_{\sigma (t)}\in R^{N\times N}$.

### 2.2. Problem Description

A collection of nonlinear MASs is considered to be composed of the N+1 agent, *N* identical nonlinear followers, and a leader who satisfies the nonlinear condition. For each follower, the dynamic equation is depicted as follows:

(1) $${\dot{x}}_{i}\left(t\right)=Ax_{i}\left(t\right)+Bu_{i}\left(t\right)+\vartheta \left(\varepsilon_{i}\left(t\right)\right)$$

Among them, $x_{i}\in R^{n}$ is the state information of the *i*-th agent; $u_{i}\in R^{m}$ is the control input of the multi-agent system; $A\in R^{n\times n}$ and $B\in R^{n\times m}$ are system matrices with corresponding dimensions; $\vartheta :R^{n}\to R^{n}$ represents an unknown nonlinear term in the follower, $\varepsilon_{i}\left(t\right)=x_{i}\left(t\right)-h_{i}\left(t\right)$, i=1,2,…,N; and $h_{i}\left(t\right)$, the vector representing the desired position configuration, will be defined later.

The nonlinear leader’s agent is described as follows:

(2) $${\dot{x}}_{0}\left(t\right)=Ax_{0}\left(t\right)+Bu_{0}\left(t\right)+\vartheta \left(\varepsilon_{0}\left(t\right)\right)$$

Among them, $x_{0}\left(t\right)$ indicates the leader’s status; $u_{0}\left(t\right)$ represents the leader’s control input; and $\vartheta :R^{n}\to R^{n}$ represents the unknown nonlinear term of the leader.

**Assumption 1.**  *ϑ(·) satisfies Lipschitz, as follows:*

(3) $$|\vartheta \left(f_{1}\right)-\vartheta \left(f_{2}\right)\left|\leq \omega \right|f_{1}-f_{2}|,\forall f_{1},f_{2}\in R^{n}$$

**Remark 1.**  *Both the follower and the leader contain nonlinear terms. However, this paper selects different nonlinear terms for the leader and follower, so (1) and (2) constitute heterogeneous nonlinear MASs.*

In order to analyze changing topology $\bar{G}$ of the leader–follower MAS, the following general assumption is given: there is a switching signal $\sigma :[t_{0},\infty )\to P$, which is a piecewise constant. P is the limited collection of all potential interconnection topologies of the MAS, and $t_{0}$ is the initial time. Defining the vertices {0,1,2,…,N} as representing all possible nodes on the graph, the set of all possible topological connection diagrams is denoted by $\left\{\bar{G}:p\in P\right\}$, and $\left\{\bar{G}:p\in P\right\}$ is the sub-atlas defined at point {0,1,2,…,N}.

**Remark 2.**  *In order to ensure that each follower tracks the leader in completing the TVF, by defining the h(t) required for a MAS, it is clear that h(t) is a function of time, meaning that the relative offset between all followers and leaders is constantly changing over time. And this paper also needs to consider that the topology is switched over time.*

**Assumption 2.**  *The pair (A,B) is stabilizable.*

**Assumption 3.**  *The leader-related vertex 0 is the global reachable point in the undirected graph $\bar{G}$.*

**Assumption 4.**  *The child graph associated with the follower is undirected, and in the union of all such subgraphs within the graph, the leader has a directed path to all followers.*

**Definition 1.**  *In the case of any arbitrarily provided initial state, if the closed-loop system satisfies the following condition:*

(4) $$\lim_{t\to \infty }\left|x_{i}\left(t\right)-x_{0}\left(t\right)-h_{i}\left(t\right)\right|=0,\;i=1,2,\ldots ,N$$

*then the heterogeneous nonlinear MASs defined by (1) and (2) satisfy the above conditions, indicating that the MASs complete the TVF control under the jointly connected topology.*

The temporal interval $\left[t_{0},\infty \right)$ is made up of bounded, non-intersecting, continuous time intervals $\left[t_{j},t_{j+1}\right)$ for j=0,1,… and $t_{0}=0$. For each separate interval $\left[t_{j},t_{j+1}\right)$, there is a non-overlapping interval sequence $\left[t_{k}^{0},t_{k}^{1}\right),\left[t_{k}^{1},t_{k}^{2}\right),\ldots ,\left[t_{k}^{m_{k}-1},t_{k}^{m_{k}}\right)$, $t_{k}=t_{k}^{0}$, $t_{k+1}=t_{k}^{m_{k}}$, satisfying $t_{k}^{j+1}-t_{k}^{j}\geq \tau$, $0\leq j\leq m_{k}-1$. $m_{k}$ is an integer greater than 0 for some integers, and a constant τ>0 is given such that the topology remains unaltered during the entire duration of the time interval $\left[t_{k}^{j},t_{k}^{j+1}\right)$, which is denoted as $G_{\sigma (t)}$. Within the time period $\left[t_{k}^{j},t_{k}^{j+1}\right)$, some or all of the communication topologies are structured and represented as $G_{kj}$ ($j=0,1,\ldots ,m_{k}-1$) and are allowed to be non-connected, as long as the joint communication topology satisfies the definitions given below.

**Definition 2.**  *The union graph is the union of graphs; the vertex set and edge set of the union graph are the unions of the vertex sets and edge sets of the component graphs. If the joint graph is connected, then the joint graph is jointly connected. If the joint graph $\left\{{\bar{G}}_{\sigma (t)}:s\in \left[t,t+T\right]\right\}$ of a multi-agent system is jointly connected, then the group of graphs is said to remain jointly connected during the time period [t,t+T], where T>0.*

The following assumptions are very important for studying the problem of switching topology.

**Assumption 5.**  *Let the joint graphs forming graphs for MASs (1) and (2) be jointly connected for each time period $\left[t_{k},t_{k+1}\right)$ (k=0,1,…).*

**Lemma 1**  ([35]). *Stays in a state of joint connectivity during the time span $\left[t_{k},t_{\left(k+1\right)}\right]$, if and only if*

$$\underset{t\in [t_{k},t_{k+1})}{⋃}l\left(\sigma \left(t\right)\right)=\left\{1,2,\ldots ,N\right\}$$

## 3. Main Results

In this section, the problem of adaptive TVF tracking control for heterogeneous nonlinear MASs with a jointly connected topology will be discussed. Therefore, the controller is designed as follows:

(5) $$\begin{array}{cc} u_{i}\left(t\right) & =K\sum_{j=1}^{N}w_{ij}\left(t\right)\eta_{ij}\left(t\right)\left(x_{i}\left(t\right)-h_{i}\left(t\right)\right.\\ \; & \;\left.-(x_{j}\left(t\right)-h_{j}\left(t\right))\right)+Kd_{i0}\left(t\right)\eta_{i0}\left(t\right)\left(x_{i}\left(t\right)-h_{i}\left(t\right)-x_{0}\left(t\right)\right)+\xi_{i}\left(t\right)\\ {\dot{\eta }}_{ij}\left(t\right) & =\kappa w_{ij}\left(t\right){\left(x_{i}\left(t\right)-h_{i}\left(t\right)-\left(x_{j}\left(t\right)-h_{j}\left(t\right)\right)\right)}^{T}\Gamma \left(x_{i}\left(t\right)-h_{i}\left(t\right)-\left(x_{j}\left(t\right)-h_{j}\left(t\right)\right)\right)\\ {\dot{\eta }}_{i0}\left(t\right) & =\partial d_{i0}\left(t\right){\left(x_{i}\left(t\right)-h_{i}\left(t\right)-x_{0}\left(t\right)\right)}^{T}\Gamma \left(x_{i}\left(t\right)-h_{i}\left(t\right)-x_{0}\left(t\right)\right) \end{array}$$

Among them, κ and *∂* denote adaptive parameter convergence factors, and κ>0, ∂>0, and $\eta_{ij}\left(t\right),\eta_{i0}\left(t\right)$ denote adaptive coupling weights between neighboring agents and adaptive coupling weights between following agents and leaders, respectively. Since the communication topology network structure is an undirected graph, $\eta_{ij}\left(t\right)=\eta_{ji}\left(t\right)$. $K\in R^{m\times n}$ is the feedback gain matrix, $\Gamma \in R^{n\times n}$ is a continuous gain matrix, and $\xi_{i}\left(t\right)$ is the input compensation in the extended feasible formation set, which can be determined by the formation feasibility condition.

Given the formation vector h(t) and the leader’s control input $u_{0}\left(t\right)$, the TVF feasibility condition for the compensating input $\xi_{i}\left(t\right)$ is derived as follows:

(6) $$Ah_{i}\left(t\right)-{\dot{h}}_{i}\left(t\right)-Bu_{0}\left(t\right)+B\xi_{i}\left(t\right)=0,\;i=1,\ldots ,N$$

**Remark 3.**  *For the heterogeneous nonlinear MASs made up of (1) and (2), if the TVF control objective needs to be satisfied, then the TVF feasibility condition of Formula (6) is very important. The coefficient matrix and the TVF vector function $h_{i}\left(t\right)$ in the nonlinear dynamic equation should satisfy Formula (6); otherwise, they need to be redesigned.*

**Theorem 1.**  *Consider that when a heterogeneous nonlinear MAS consists of (1) and (2), Assumptions 1–5 hold, and the required TVF configuration vector satisfies the TVF tracking feasibility condition (6), then, under jointly connected topologies, the fully distributed adaptive TVF tracking control problem can be resolved. The following LMI represents the feedback gain matrix and the continuous gain matrix:*

(7) $$\left[\begin{array}{cc} AP+PA^{T}-\theta BB^{T}+\gamma^{2}I & P\\ P & -I \end{array}\right]<0$$

A matrix P>0 can be obtained, and the parameter θ>0. And feedback gain matrix is given by $K=-B^{T}P^{-1}$, and the continuous gain matrix is $\Gamma =P^{-1}BB^{T}P^{-1}$.

**Proof of Theorem 1.**  Bringing each follower agent (1) into the controller (5):

(8) $$\begin{array}{cc} {\dot{x}}_{i}\left(t\right) & =Ax_{i}\left(t\right)+Bu_{i}\left(t\right)\\ \; & =Ax_{i}\left(t\right)+\vartheta \left(\varepsilon_{i}\left(t\right)\right)+B\xi_{i}\left(t\right)\\ \; & \;-BB^{T}P^{-1}\left[\sum_{j=1}^{N}w_{ij}\eta_{ij}\left(t\right)\left(x_{i}\left(t\right)-h_{i}\left(t\right)-\left(x_{j}\left(t\right)-h_{j}\left(t\right)\right)\right)\right.\\ \; & \;\left.+d_{i0}\eta_{i0}\left(t\right)\left(x_{i}\left(t\right)-h_{i}\left(t\right)-x_{0}\left(t\right)\right)\right] \end{array}$$

Let $\zeta_{i}\left(t\right)=x_{i}\left(t\right)-h_{i}\left(t\right)-x_{0}\left(t\right)$, $\zeta_{i}\left(t\right)$ denote the TVF tracking error for each agent, $\zeta \left(t\right)={\left[\zeta_{1}\left(t\right),\zeta_{2}\left(t\right),\zeta_{3}\left(t\right)\ldots \zeta_{N}\left(t\right)\right]}^{T}$, and make ${\dot{\zeta }}_{i}\left(t\right)={\dot{x}}_{i}\left(t\right)-{\dot{h}}_{i}\left(t\right)-{\dot{x}}_{0}\left(t\right)$ available:

(9) $$\begin{array}{cc} {\dot{\zeta }}_{i}\left(t\right) & =A\zeta_{i}\left(t\right)+\vartheta \left(\varepsilon_{i}\left(t\right)\right)-\vartheta \left(x_{0}\left(t\right)\right)\\ \; & \;-BB^{T}P^{-1}\left[\sum_{j=1}^{N}w_{ij}\eta_{ij}\left(t\right)\left(\zeta_{i}\left(t\right)-\zeta_{j}\left(t\right)\right)+d_{i0}\eta_{i0}\left(t\right)\zeta_{i}\left(t\right)\right]\\ \; & \;+Ah_{i}\left(t\right)-{\dot{h}}_{i}\left(t\right)-Bu_{0}\left(t\right)+B\xi_{i}\left(t\right)\\ {\dot{\tilde{\eta }}}_{ij} & =\kappa w_{ij}\left(t\right){\left(\zeta_{i}\left(t\right)-\zeta_{j}\left(t\right)\right)}^{T}\Gamma \left(\zeta_{i}\left(t\right)-\zeta_{j}\left(t\right)\right)\\ {\dot{\tilde{\eta }}}_{i0} & =\partial d_{i0}\left(t\right)\zeta_{i}^{⊺}\left(t\right)\Gamma \zeta_{i}\left(t\right) \end{array}$$

Among them, $\eta_{ij}={\tilde{\eta }}_{ij}+\alpha$, $\eta_{i0}={\tilde{\eta }}_{i0}+\alpha$, α is a positive number. Since the heterogeneous nonlinear systems (1) and (2) need to satisfy the TVF feasibility conditions, the following equation can be obtained from Equation (6) to Equation (9):

(10) $${\dot{\zeta }}_{i}\left(t\right)=A\zeta_{i}\left(t\right)+\vartheta \left(\varepsilon_{i}\left(t\right)\right)-\vartheta \left(x_{0}\left(t\right)\right)-BB^{T}P^{-1}\left[\sum_{j=1}^{N}w_{ij}\eta_{ij}\left(t\right)\left(\zeta_{i}\left(t\right)-\zeta_{j}\left(t\right)\right)+d_{i0}\eta_{i0}\left(t\right)\zeta_{i}\left(t\right)\right]$$

Consider the alternative Lyapunov function:

(11) $$V\left(t\right)=\sum_{i=1}^{N}\zeta_{i}^{T}\left(t\right)P^{-1}\zeta_{i}\left(t\right)+\sum_{i=1}^{N}\sum_{j=1,j\neq i}^{N}\frac{{\tilde{\eta }}_{ij}^{2}}{2\kappa }+\sum_{i=1}^{N}\frac{{\tilde{\eta }}_{i0}^{2}}{\partial }$$

We obtain the derivative of Equation (11):

(12) $$\dot{V}\left(t\right)=2\sum_{i=1}^{N}\zeta_{i}^{1}\left(t\right)P^{-1}{\dot{\zeta }}_{i}\left(t\right)+\sum_{i=1}^{N}\sum_{j=1,j\neq i}^{N}\frac{{\tilde{\eta }}_{ij}}{\kappa }{\tilde{\eta }}_{ij}+2\sum_{i=1}^{N}\frac{{\tilde{\eta }}_{i0}}{\partial }{\tilde{\eta }}_{i0}$$

Substituting Equations (9) and (10) into (12) yields the following:

(13) $$\begin{array}{cc} \dot{V}\left(t\right) & =2\sum_{i=1}^{N}\zeta_{i}^{T}\left(t\right)P^{-1}\left(A\zeta_{i}\left(t\right)+\vartheta \left(\varepsilon_{i}\left(t\right)\right)-\vartheta \left(x_{0}\left(t\right)\right)\right.\\ \; & \;-BB^{T}P^{-1}\left[\sum_{j=1}^{N}w_{ij}\eta_{ij}\left(t\right)\left(\zeta_{i}\left(t\right)-\zeta_{j}\left(t\right)\right)+d_{i0}\eta_{i0}\left(t\right)\zeta_{i}\left(t\right)\right])\\ \; & \;+\sum_{i=1}^{N}\sum_{j=1,j\neq i}^{N}\left(\eta_{ij}-\alpha \right)w_{ij}\left(t\right){\left(\zeta_{i}\left(t\right)-\zeta_{j}\left(t\right)\right)}^{T}\Gamma \left(\zeta_{i}\left(t\right)-\zeta_{j}\left(t\right)\right)\\ \; & \;+2\sum_{i=1}^{N}\left(\eta_{i0}-\alpha \right)d_{i0}\left(t\right)\zeta_{i}^{T}\left(t\right)\Gamma \zeta_{i}\left(t\right) \end{array}$$

Formula (13) is divided into the following:

(14) $$\begin{array}{cc} \dot{V}\left(t\right) & =\sum_{i=1}^{N}\zeta_{i}^{T}\left(t\right)\left(P^{-1}A+A^{T}P^{-1}\right)\zeta_{i}\left(t\right)+2\sum_{i=1}^{N}\zeta_{i}^{T}\left(t\right)P^{-1}\left(\vartheta \left(\varepsilon_{i}\left(t\right)\right)-\vartheta \left(x_{0}\left(t\right)\right)\right)\\ & -2\sum_{i=1}^{N}\zeta_{i}^{T}\left(t\right)P^{-1}BB^{T}P^{-1}\left[\sum_{j=1}^{N}w_{ij}\eta_{ij}\left(t\right)\left(\zeta_{i}\left(t\right)-\zeta_{j}\left(t\right)\right)+d_{i0}\eta_{i0}\left(t\right)\zeta_{i}\left(t\right)\right]\\ & +\sum_{i=1}^{N}\sum_{j=1,j=i}^{N}\left(\eta_{ij}-\alpha \right)w_{ij}\left(t\right){\left(\zeta_{i}\left(t\right)-\zeta_{j}\left(t\right)\right)}^{T}\Gamma \left(\zeta_{i}\left(t\right)-\zeta_{j}\left(t\right)\right)+2\sum_{i=1}^{N}\left(\eta_{i0}-\alpha \right)d_{i0}\left(t\right)\zeta_{i}^{T}\left(t\right)\Gamma \zeta_{i}\left(t\right) \end{array}$$

From Lipschitz condition (3), the following can be stated:

(15) $$\begin{array}{cc} 2\sum_{i=1}^{N}\zeta_{i}^{T}\left(t\right)P^{-1}\left(\vartheta \left(\varepsilon_{i}\left(t\right)\right)-\vartheta \left(x_{0}\left(t\right)\right)\right) & \leq 2\gamma \sum_{i=1}^{N}\zeta_{i}^{T}\left(t\right)P^{-1}∥\varepsilon_{i}\left(t\right)-x_{0}\left(t\right)∥\\ & \leq 2\gamma \sum_{i=1}^{N}∥P^{-1}\zeta_{i}\left(t\right)∥∥\zeta_{i}\left(t\right)∥\\ & \leq \sum_{i=1}^{N}\zeta_{i}^{T}\left(t\right)\left[\gamma^{2}{\left(P^{-1}\right)}^{2}+I\right]\zeta_{i}\left(t\right) \end{array}$$

Looking at controller (5), it can be seen that $\eta_{ij}\left(t\right)=\eta_{ji}\left(t\right),\forall t\geq 0$. And by inserting $\Gamma =P^{-1}BB^{T}P^{-1}$ into it, we get the following:

(16) $$\begin{array}{cc} & \sum_{i=1}^{N}\sum_{j=1,j\neq i}^{N}\left(\eta_{ij}-\alpha \right)w_{ij}\left(t\right){\left(\zeta_{i}\left(t\right)-\zeta_{j}\left(t\right)\right)}^{⊺}\Gamma \left(\zeta_{i}\left(t\right)-\zeta_{j}\left(t\right)\right)\\ & =\sum_{i=1}^{N}\sum_{j=1,j\neq i}^{N}\left(\eta_{ij}-\alpha \right)w_{ij}\left(t\right){\left(\zeta_{i}\left(t\right)-\zeta_{j}\left(t\right)\right)}^{T}P^{-1}BB^{T}P^{-1}\left(\zeta_{i}\left(t\right)-\zeta_{j}\left(t\right)\right)\\ & =2\sum_{i=1}^{N}\sum_{j=1,j\neq i}^{N}\left(\eta_{ij}-\alpha \right)w_{ij}\left(t\right)\zeta_{i}^{T}\left(t\right)P^{-1}BB^{T}P^{-1}\left(\zeta_{i}\left(t\right)-\zeta_{j}\left(t\right)\right) \end{array}$$

Let ${\hat{\zeta }}_{i}\left(t\right)=P^{-1}\zeta_{i}\left(t\right)$, $\hat{\zeta }\left(t\right)={\left[{\hat{\zeta }}_{1}\left(t\right),{\hat{\zeta }}_{2}\left(t\right),{\hat{\zeta }}_{3}\left(t\right)\ldots {\hat{\zeta }}_{N}\left(t\right)\right]}^{T}$. Bringing (15) and (16) into (14) yields the following:

(17) $$\begin{array}{cc} \dot{V}\left(t\right) & \leq \sum_{i=1}^{N}{\hat{\zeta }}_{i}^{T}\left(t\right)\left(AP+PA^{T}\right){\hat{\zeta }}_{i}\left(t\right)+\sum_{i=1}^{N}{\hat{\zeta }}_{i}^{T}\left(t\right)\left[\gamma^{2}I+P^{2}\right]{\hat{\zeta }}_{i}\left(t\right)\\ & -\sum_{i-1}^{N}\sum_{j-1,j=i}^{N}2\alpha w_{ij}\left(t\right){\hat{\zeta }}_{i}^{T}\left(t\right)BB^{T}\left({\hat{\zeta }}_{i}\left(t\right)-{\hat{\zeta }}_{j}\left(t\right)\right)-\sum_{i=1}^{N}2\alpha d_{i0}\left(t\right){\hat{\zeta }}_{i}^{T}\left(t\right)BB^{T}{\hat{\zeta }}_{i}\left(t\right) \end{array}$$

If $H_{\sigma (t)}=L_{\sigma (t)}+D_{\sigma (t)}$ is satisfied, Formula (17) can be rewritten as follows:

(18) $$\dot{V}\left(t\right)=\sum_{i=1}^{N}{\hat{\zeta }}_{i}^{T}\left(t\right)\left(\left[AP+PA^{T}+\gamma^{2}I+P^{2}\right]-2\alpha \sum_{i=1}^{N}\sum_{j=1,j\neq i}^{N}H_{ij}^{\sigma (t)}BB^{T}\right){\hat{\zeta }}_{i}\left(t\right)$$

Writing Equation (18) in a compact form gives the following:

(19) $$\dot{V}\left(t\right)={\hat{\zeta }}^{T}\left(t\right)\left(I_{N}⊗\left[AP+PA^{T}+\gamma^{2}I+P^{2}\right]-2\alpha H_{\sigma (t)}⊗BB^{T}\right)\hat{\zeta }\left(t\right)$$

Because the matrix $H_{\sigma (t)}$ is symmetric, an orthogonal matrix $T_{\sigma (t)}$ can always be found at any non-switching moment, such that $H_{\sigma (t)}$ is transformed into a diagonal form:

(20) $$T_{p}H_{\sigma (t)}T_{p}^{T}=\Lambda_{\sigma (t)}=diag\left(\lambda_{\sigma (t)}^{\pi_{p}\left(1\right)},\lambda_{\sigma (t)}^{\pi_{p}\left(2\right)},\ldots ,\lambda_{\sigma (t)}^{\pi_{p}\left(n\right)}\right)$$

Among them, $\pi_{p}$ is some permutation of the set {1,2,…,N}. Let $\tilde{\zeta }\left(t\right)=\left(T_{\sigma (t)}⊗I_{n}\right)\hat{\zeta }\left(t\right)$. After substitution, the following expression is obtained:

(21) $$\begin{array}{cc} \dot{V}\left(t\right) & \leq {\tilde{\varsigma }}^{T}\left(t\right)\left(I_{N}⊗\left[\begin{array}{cc} AP+PA^{T}+\gamma^{2}I & -P\\ P & I \end{array}\right]-2\alpha \Lambda_{\sigma (t)}⊗BB^{T}\right)\tilde{\zeta }\left(t\right)\\ \; & \leq \sum_{i=1}^{N}{\tilde{\zeta }}_{i}^{T}\left(t\right)\left(\left[\begin{array}{cc} AP+PA^{T}+\gamma^{2}I & -P\\ P & I \end{array}\right]-2\alpha \lambda_{1}BB^{T}\right){\tilde{\zeta }}_{i}\left(t\right)\\ \; & \leq \sum_{i=1}^{N}{\tilde{\zeta }}_{i}^{T}\left(t\right)\left[\begin{array}{cc} AP+PA^{T}-2\alpha \lambda_{1}BB^{T}+\gamma^{2}I & P\\ P & -I \end{array}\right]{\tilde{\zeta }}_{i}\left(t\right) \end{array}$$

As long as an appropriate α is selected, satisfying $2\alpha \lambda_{i}>\theta ,i=1,2\ldots N$, (21) is converted into the following:

(22) $$\begin{array}{cc} \dot{V}\left(t\right) & \leq \sum_{i=1}^{N}{\tilde{\zeta }}_{i}^{T}\left(t\right)\left[\begin{array}{cc} AP+PA^{T}-\theta BB^{T}+\gamma^{2}I & P\\ P & -I \end{array}\right]{\tilde{\zeta }}_{i}\left(t\right)\\ & \leq -\sum_{i=1}^{N}{\tilde{\zeta }}_{i}^{T}\left(t\right){\tilde{\zeta }}_{i}\left(t\right)\leq 0 \end{array}$$

For any i∈l(σ(t)), from the above, it becomes apparent that $\dot{V}\leq 0$ exists, and then $\lim_{t\to \infty }V_{1}\left(t\right)$ exists. In light of Cauchy’s convergence criterion, for any E>0, there always exists a positive number $M_{\mu }$, so that for any $m>M_{\mu }$, we have the following:

(23) $$\left|V\left(\tilde{\zeta }\left(t_{k+1}\right)\right)-V\left(\tilde{\zeta }\left(t_{k}\right)\right)\right|=\left|\int_{t_{k}}^{t_{k+1}}\dot{V}\left(\tilde{\zeta }\left(t\right)\right)dt\right|<\Xi$$

We can rewrite Equation (23) as follows:

(24) $$-\Xi <\sum_{j=0}^{m_{k}-1}\int_{t_{k}}^{t_{k+1}}\dot{V}\left(\tilde{\zeta }\left(t\right)\right)dt\leq -\sum_{j=0}^{m_{k}-1}\int_{t_{k}^{j}}^{t_{k}^{j+1}}{\tilde{\zeta }}^{T}\left(t\right)\tilde{\zeta }\left(t\right)dt\leq -\sum_{j=0}^{m_{k}-1}\int_{t_{k}^{j}}^{t_{k}^{j}+\tau }{\tilde{\zeta }}^{T}\left(t\right)\tilde{\zeta }\left(t\right)dt$$

Therefore, for any $j=0,1,\ldots ,m_{k}-1$; we reformulate Equation (24) as follows:

(25) $$\begin{array}{cc} \; & \int_{t_{k}^{0}}^{t_{k}^{0}+\tau }\sum_{i\in l(\sigma \left(t_{k}^{0}\right))}{\tilde{\zeta }}_{i}^{T}\left(s\right){\tilde{\zeta }}_{i}\left(s\right)ds+\int_{t_{k}^{1}}^{t_{k}^{1}+\tau }\sum_{i\in l(\sigma \left(t_{k}^{1}\right))}{\tilde{\zeta }}_{i}^{T}\left(s\right){\tilde{\zeta }}_{i}\left(s\right)ds+\ldots \\ & +\int_{t_{k}^{m_{k-1}}}^{t_{k}^{m_{k-1}}+\tau }\sum_{i\in l(\sigma \left(t_{k}^{m_{k}-1}\right))}{\tilde{\zeta }}_{i}^{T}\left(s\right){\tilde{\zeta }}_{i}\left(s\right)ds<\Xi \end{array}$$

This means the following:

(26) $$\lim_{t\to \infty }\int_{t}^{t+\tau }\sum_{i\in l(\sigma \left(t_{k}^{j}\right))}{\tilde{\zeta }}_{i}^{⊤}\left(s\right){\tilde{\zeta }}_{i}\left(s\right)ds=0$$

This is equal to the following:

(27) $$\lim_{t\to \infty }\int_{t}^{t+\tau }\left[\sum_{i\in l(\sigma \left(t_{k}^{0}\right))}{\tilde{\zeta }}_{i}^{T}\left(s\right){\tilde{\zeta }}_{i}\left(s\right)+\sum_{i\in l(\sigma \left(t_{k}^{1}\right))}{\tilde{\zeta }}_{i}^{T}\left(s\right){\tilde{\zeta }}_{i}\left(s\right)+\ldots +\sum_{i\in l(\sigma \left(t_{k}^{m_{k}-1}\right))}{\tilde{\zeta }}_{i}^{T}\left(s\right){\tilde{\zeta }}_{i}\left(s\right)\right]ds=0$$

According to the slave Lemma 1, because of the joint connectivity during $\left[t_{k},t_{\left(k+1\right)}\right]$, Equation (27) can be rewritten as follows:

(28) $$\lim_{t\to \infty }\int_{t}^{t+\tau }\left[\sum_{i=1}^{n}b_{i}{\tilde{\zeta }}_{i}^{T}\left(s\right){\tilde{\zeta }}_{i}\left(s\right)\right]ds=0$$

□

Among them, $b_{1},b_{2},\ldots b_{n}$ are positive integers. So, we have $\lim_{t\to \infty }\sum_{i=1}^{n}b_{i}{\tilde{\zeta }}_{i}^{T}\left(t\right){\tilde{\zeta }}_{i}\left(t\right)=0$, and it is not hard to see $\lim_{t\to \infty }{\tilde{\zeta }}_{i}\left(t\right)=0$, so $\lim_{t\to \infty }\zeta_{i}\left(t\right)=0$. Thus, under the jointly connected topology with controller (5), the heterogeneous nonlinear MASs composed of (1) and (2) can realize the TVF tracking control.

**Remark 4.**  *Under controller (5), multi-agent systems enable each agent to fully access the states of its local neighbors and adaptively adjust errors through adaptive coupling weights, without relying on global information. However, in practical multi-agent systems, communication delays and packet losses are inevitable. Communication delays may cause the coupling weights to be adjusted based on outdated neighbor states, thereby triggering weight oscillations and reducing the convergence speed; packet losses can lead to intermittent interruptions in weight updates, degrading the overall consensus performance.*

## 4. Numerical Simulation

**Figure 1:**
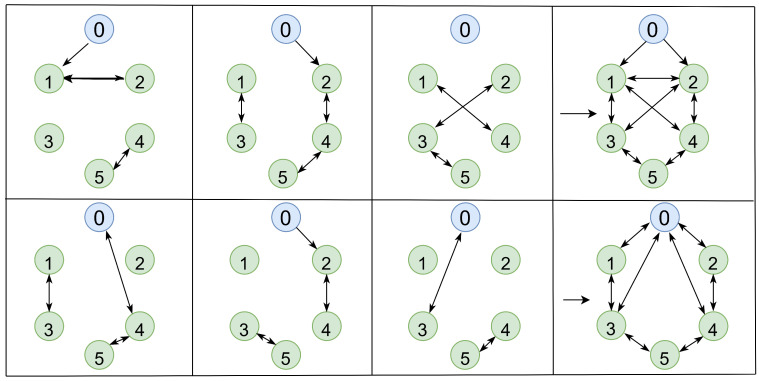
Diagram of possible communication topologies for the agent system.

**Figure 2:**
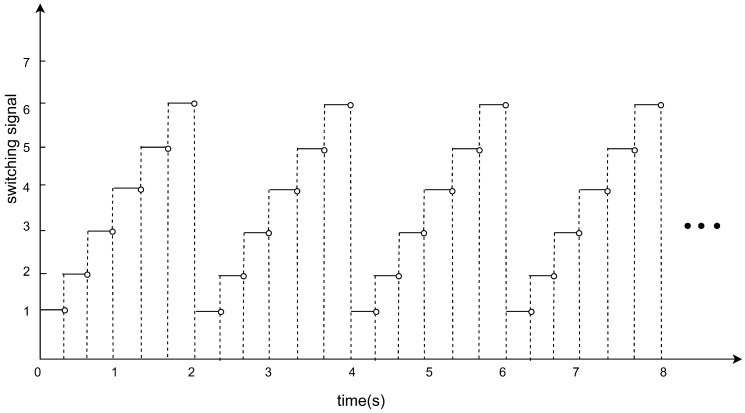
Switching signal.

This section validates the theory. TVF tracking control is achieved under controller (5), according to Equations (6) and (7). The jointly connected topology is illustrated in Figure 1, which shows all possible topologies $\left\{{\bar{G}}_{1},{\bar{G}}_{2},{\bar{G}}_{3},{\bar{G}}_{4},{\bar{G}}_{5},{\bar{G}}_{6}\right\}$. All possible communication topology diagrams are switched periodically in order of ${\bar{G}}_{1}\to {\bar{G}}_{2}\to {\bar{G}}_{3}\to {\bar{G}}_{4}\to {\bar{G}}_{5}\to {\bar{G}}_{6}\to {\bar{G}}_{1}\cdots$. As shown in Figure 2, a switching period of 2 s is divided into six switching times, and a communication structure diagram is used during each switching time.

**Figure 3:**
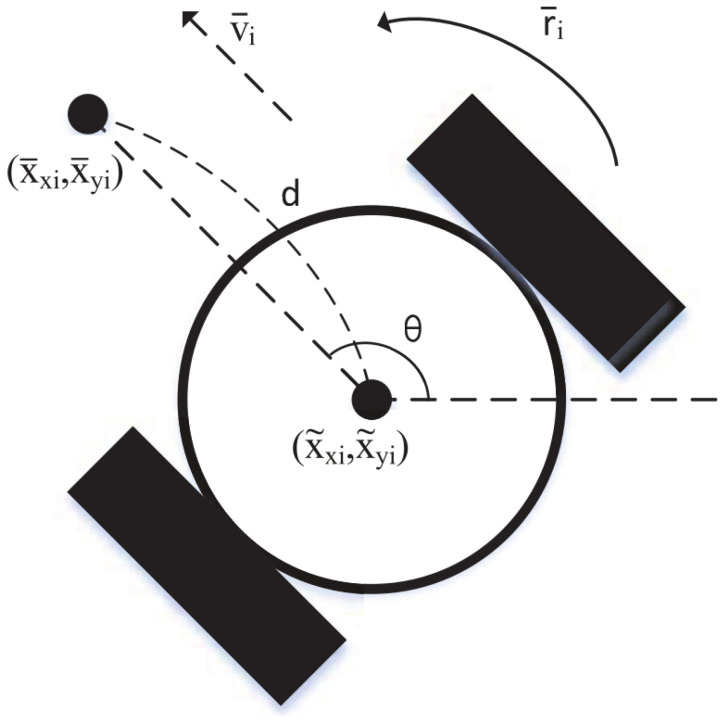
Structure diagram of the nonholonomic mobile robot.

The simulation considers the non-holonomic mobile robot model as shown in Figure 3. All intelligent bodies have the same structure and motion model, and are described by the following kinematic equations: ${\dot{\tilde{x}}}_{xi}={\bar{v}}_{i}\cos\theta_{i},{\dot{\tilde{x}}}_{yi}={\bar{v}}_{i}\sin\theta_{i},{\dot{\theta }}_{i}={\bar{r}}_{i}$. Among them, $\left({\tilde{x}}_{xi},{\tilde{x}}_{yi}\right),{\bar{v}}_{i},\theta_{i},{\bar{r}}_{i}$, respectively, represent the center position, linear velocity, heading angle and rotational velocity of the *i*-th robot. For the analysis of non-complete mobile robots, by analogy with Reference [36], a fixed point deviating from the center of the wheel is taken as $\left({\bar{x}}_{xi},{\bar{x}}_{yi}\right)$, which is taken as the inertial position of the *i*-th non-holonomic mobile robot, where $\left[\begin{array}{c} {\bar{x}}_{xi}\\ {\bar{x}}_{yi} \end{array}\right]=\left[\begin{array}{c} {\tilde{x}}_{xi}\\ {\tilde{x}}_{yi} \end{array}\right]+d\left[\begin{array}{c} \cos\theta_{i}\\ \sin\theta_{i} \end{array}\right]$. Secondly, we can obtain $\left[\begin{array}{c} {\dot{\bar{x}}}_{xi}\\ {\dot{\bar{x}}}_{yi} \end{array}\right]=\left[\begin{array}{cc} \cos\theta_{i} & -d\sin\theta_{i}\\ \sin\theta_{i} & d\cos\theta_{i} \end{array}\right]\left[\begin{array}{c} {\bar{v}}_{i}\\ {\bar{r}}_{i} \end{array}\right]$, where $\left[\begin{array}{c} {\bar{v}}_{i}\\ {\bar{r}}_{i} \end{array}\right]=\left[\begin{array}{cc} \cos\theta_{i} & \sin\theta_{i}\\ -\sin\theta_{i}/d & \cos\theta_{i}/d \end{array}\right]\left[\begin{array}{c} {\bar{v}}_{xi}\\ {\bar{v}}_{yi} \end{array}\right],d\neq 0$. Finally, we define ${\bar{v}}_{xi}=u_{xi},{\bar{v}}_{yi}=u_{yi}$, which, respectively, represent the linear velocity components along the X and Y directions.

Using incomplete feedback linearization, the kinematic model is converted into a dynamic equation. Consider a multi-agent system consisting of five agents and one leader. The state of each agent is defined as $x_{i}={\left[{\bar{x}}_{xi}^{T},{\bar{v}}_{xi}^{T},{\bar{x}}_{yi}^{T},{\bar{v}}_{yi}^{T}\right]}^{T}$, and the control input is $u_{i}={\left[u_{xi}^{T},u_{yi}^{T}\right]}^{T}$. Assume that the coefficient matrices (1) and (2) of the system are as follows:

$$A=\left[\begin{array}{cccc} 0 & 1 & 0 & 0\\ 0 & 0 & 0 & 0\\ 0 & 0 & 0 & 1\\ 0 & 0 & 0 & 0 \end{array}\right]\;B=\left[\begin{array}{cc} 0 & 0\\ 1 & 0\\ 0 & 0\\ 0 & 1 \end{array}\right]$$

The nonlinear parameter $\vartheta (\varepsilon_{i}\left(t\right))$ of the follower is

$$\left[\begin{array}{c} 0.1\cos(\left(x_{i1}\left(t\right)-h_{i1}\left(t\right)\right)\\ 0.1\cos(x_{i2}\left(t\right)-h_{i2}\left(t\right))\\ 0.1\cos(x_{i3}\left(t\right)-h_{i3}\left(t\right))\\ 0.1\cos(x_{i4}\left(t\right)-h_{i4}\left(t\right)) \end{array}\right]$$

The leader’s nonlinear parameter $\vartheta (x_{0}\left(t\right))$ is

$${\left[0.1\cos\left(x_{01}\left(t\right)\right),0.1\cos\left(x_{02}\left(t\right)\right),0.1\cos\left(x_{03}\left(t\right)\right),0.1\cos\left(x_{04}\left(t\right)\right)\right]}^{T}$$

The follower agent needs to complete the regular pentagonal TVF tracking, and the desired TVF configuration direction is as follows:

$$h_{i}\left(t\right)=\left[\begin{array}{c} \sin\left(t+\frac{2(i-1)}{5}\pi \right)\\ \cos\left(t+\frac{2(i-1)}{5}\pi \right)\\ \cos\left(t+\frac{2(i-1)}{5}\pi \right)\\ -\sin\left(t+\frac{2(i-1)}{5}\pi \right) \end{array}\right]i=1,2,3,4$$

The formation compensation vector $\xi_{i}\left(t\right)$ calculated according to Formula (6) is as follows:

$$\xi_{i}\left(t\right)=\left[\begin{array}{c} -2\sin\left(t+\frac{2(i-1)}{5}\pi \right)\\ -2\cos\left(t+\frac{2(i-1)}{5}\pi \right) \end{array}\right],\;i=1,2,3,4$$

Given θ=25,γ=0.1, the feedback gain matrix $K=-B^{⊤}P^{-1}$ and the continuous gain matrix $\Gamma =P^{-1}BB^{⊤}P^{-1}$ are designed by solving Equation (7):

$$P=\left[\begin{array}{cccc} 0.1397 & -0.2691 & 0.0000 & 0.0000\\ -0.2691 & 1.5654 & 0.0000 & 0.0000\\ 0.0000 & 0.0000 & 0.1397 & -0.2691\\ 0.0000 & 0.0000 & -0.2691 & 1.5654 \end{array}\right]$$

$$K=\left[\begin{array}{cccc} -1.8409 & -0.9553 & 0.0000 & 0.0000\\ 0.0000 & 0.0000 & -1.8409 & -0.9553 \end{array}\right]$$

$$\Gamma =\left[\begin{array}{cccc} 3.3888 & 1.7587 & 0.0000 & 0.0000\\ 1.7587 & 0.9127 & 0.0000 & 0.0000\\ 0.0000 & 0.0000 & 3.3888 & 1.7587\\ 0.0000 & 0.0000 & 1.7587 & 0.9127 \end{array}\right]$$

The initial states of the leader’s adaptive coupling weights are set as $c_{10}\left(0\right)=20$, $c_{20}\left(0\right)=10$, $c_{30}\left(0\right)=20,\;c_{40}\left(0\right)=10,\;c_{50}\left(0\right)=10$. The initial values of the followers’ adaptive coupling weights can be arbitrarily assigned as $c_{ij}\left(0\right)=c_{ji}\left(0\right),\;i,j=1,2,\ldots ,N$. The adaptive coupling weight convergence factor is κ=100, and the parameter is ∂=150.

**Figure 4:**
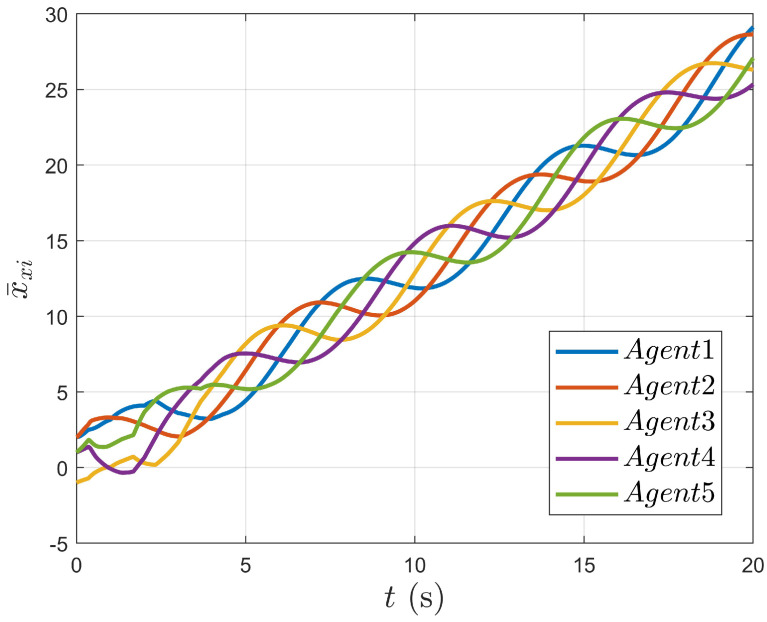

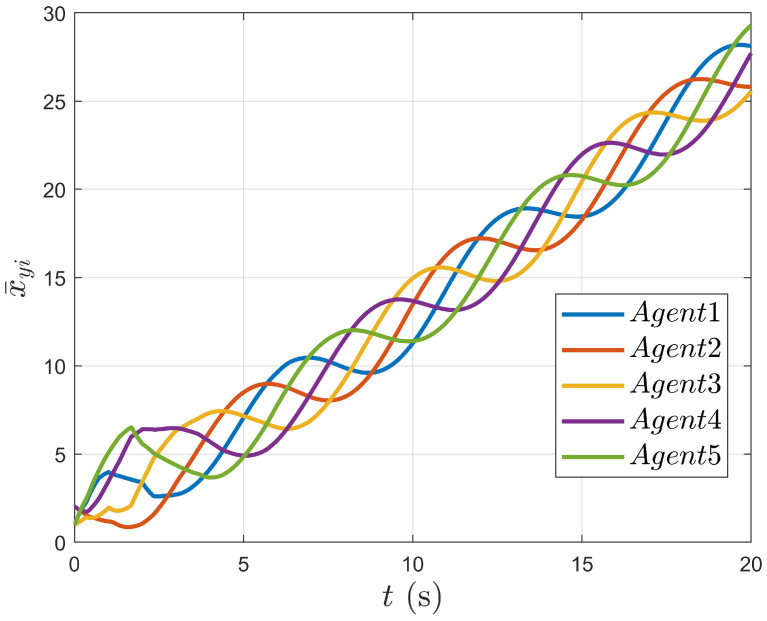
The changes in the agent’s position in the X and Y directions over time.

**Figure 5:**
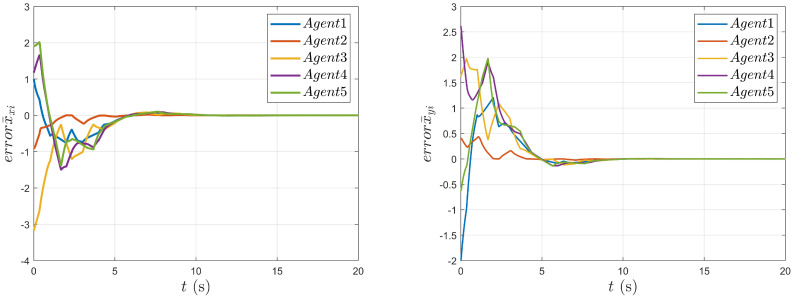
Tracking error of the agent’s position states.

**Figure 6:**
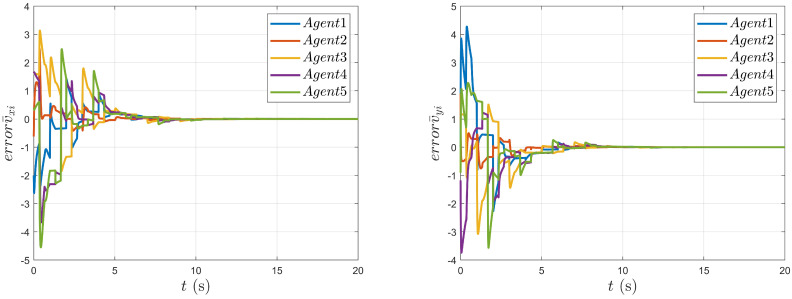
Tracking error of the agent’s speed states.

As shown in Figure 4, it can be seen that the position movements of the nonholonomic mobile robot in the X and Y directions change with time. As shown in Figure 5 and Figure 6, the tracking errors of position and velocity in both the X and Y directions gradually approach zero over time, respectively.

**Figure 7:**
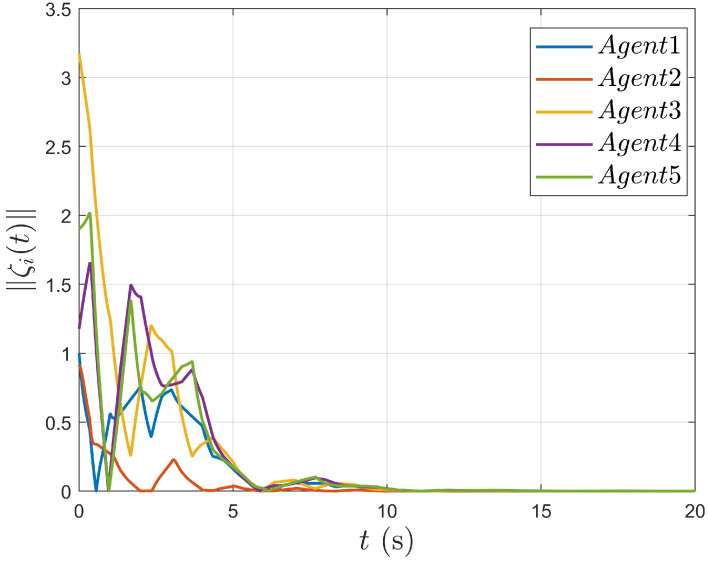
Agent’s formation error.

As shown in Figure 7, the TVF error converges to zero over time, demonstrating the stability of the MAS described by (1) and (2) in completing the TVF process under controller (5).

**Figure 8:**
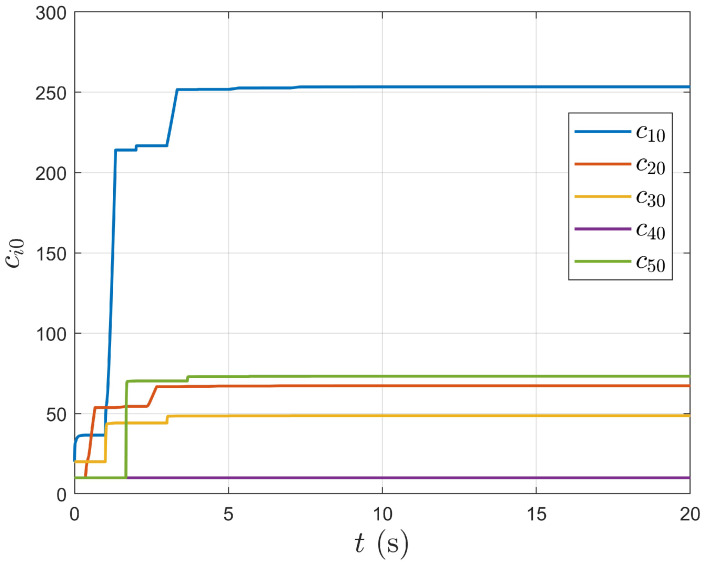
Adaptive weight curve between the leader and follower.

**Figure 9:**
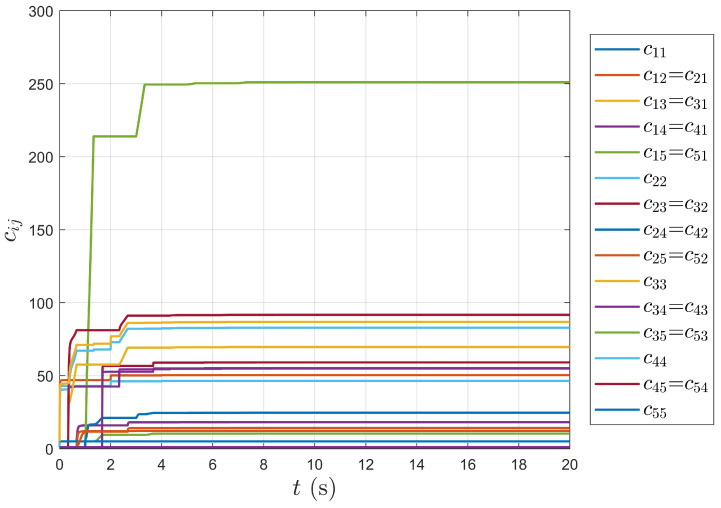
Adaptive weight curve between followers.

As shown in Figure 8 and Figure 9, the adaptive weight curves converge to fixed values over time, showing that the proposed controller (5) achieves a fully distributed system. The formation objective is completed by automatically adjusting the relative errors between agents through the adaptive weights.

**Figure 10:**
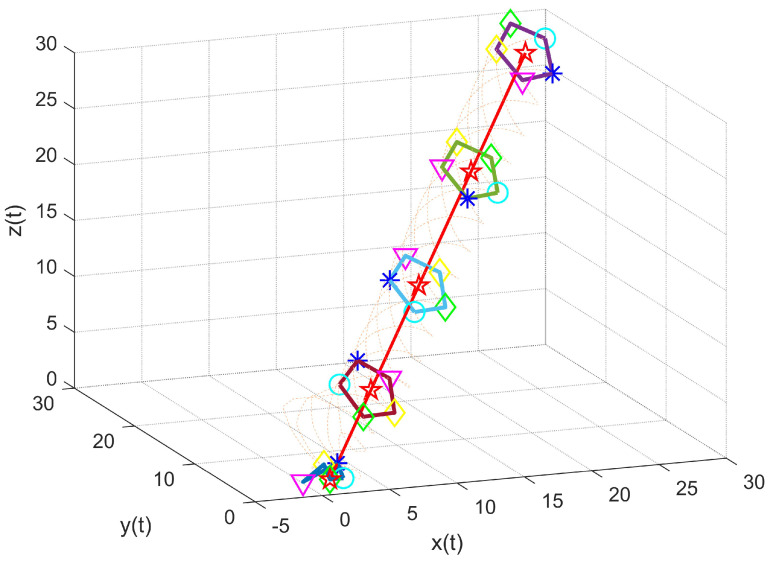
Time−varying formation trajectory of multi-agent systems.

**Figure 11:**
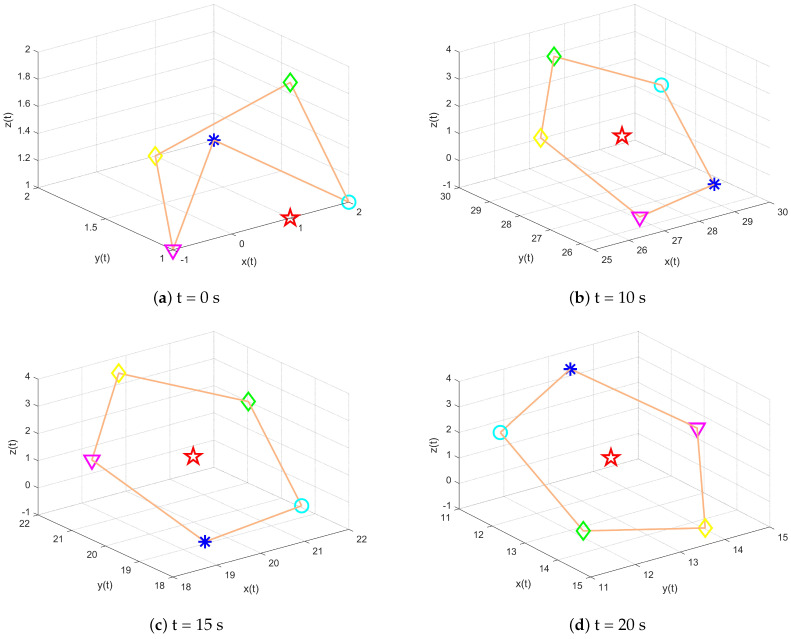
Multi−agent system formation state at each moment.

As shown in Figure 10, under the jointly connected topology, a heterogeneous MAS changes in time at any position (at the beginning) and finally completes the time-varying formation target. Figure 11 shows snapshots of the system state at various time intervals, in which the five follower agents are represented by a circle, green diamond, asterisk, triangle, and yellow diamond, respectively, while the leader is represented by a five-pointed star. The five agents complete the TVF by rotating at an angular speed of 1 rad/s.

## 5. Conclusions

This paper addresses the problem of time-varying formation-tracking control for heterogeneous nonlinear multi-agent systems with jointly connected topologies. The heterogeneous system consists of one leader and multiple followers with nonlinear terms that differ from those of the leader, and the communication topology of the entire agent system changes over time. The time-varying formation tracking is achieved under a jointly connected topological graph. A distributed adaptive controller is proposed for the heterogeneous nonlinear system, which adaptively adjusts errors through adaptive coupling weights. A stability analysis framework based on Riccati inequalities is established, and combined with the Lyapunov function method, the asymptotic stability of the system under jointly connected topologies is rigorously proven. The proof process correlates the switching frequency of time-varying topologies with the Lipschitz constants of heterogeneous nonlinear terms, breaking through the limitations of traditional stability analysis under time-invariant topologies. This provides theoretical support for cooperative control of multi-agent systems in dynamic communication environments and verifies that heterogeneous nonlinear systems can achieve time-varying formation tracking using the proposed method. In future research, we will consider issues such as system instability caused by delays or packet loss rates exceeding specific thresholds.

## Data Availability

Data are contained within the article.
